# Experimental Investigation of the Performance of a Hybrid Self-Healing System in Porous Asphalt under Fatigue Loadings

**DOI:** 10.3390/ma14123415

**Published:** 2021-06-20

**Authors:** Shi Xu, Xueyan Liu, Amir Tabaković, Erik Schlangen

**Affiliations:** 1 Hubei Key Laboratory of Roadway Bridge and Structure Engineering, Wuhan University of Technology, Wuhan 430070, China; 2 Civil Engineering and Geosciences, Delft University of Technology, 2628CN Delft, The Netherlands; x.liu@tudelft.nl (X.L.); amir.tabakovic@TUDublin.ie (A.T.); Erik.Schlangen@tudelft.nl (E.S.); 3 Centre for Research in Engineering Surface Technology (CREST), Technological University Dublin, D08CKP1 Dublin, Ireland; 4 School of Civil Engineering, University College Dublin, D04K3H4 Dublin, Ireland

**Keywords:** self-healing asphalt, fatigue life, induction heating, calcium alginate capsules, combined healing system

## Abstract

Self-healing asphalt, which is designed to achieve autonomic damage repair in asphalt pavement, offers a great life-extension prospect and therefore not only reduces pavement maintenance costs but also saves energy and reduces CO_2_ emissions. The combined asphalt self-healing system, incorporating both encapsulated rejuvenator and induction heating, can heal cracks with melted binder and aged binder rejuvenation, and the synergistic effect of the two technologies shows significant advantages in healing efficiency over the single self-healing method. This study explores the fatigue life extension prospect of the combined healing system in porous asphalt. To this aim, porous asphalt (PA) test specimens with various healing systems were prepared, including: (i) the capsule healing system, (ii) the induction healing system, (iii) the combined healing system and (iv) a reference system (without extrinsic healing). The fatigue properties of the PA samples were characterized by an indirect tensile fatigue test and a four-point bending fatigue test. Additionally, a 24-h rest period was designed to activate the built-in self-healing system(s) in the PA. Finally, a damaging and healing programme was employed to evaluate the fatigue damage healing efficiency of these systems. The results indicate that all these self-healing systems can extend the fatigue life of porous asphalt, while in the combined healing system, the gradual healing effect of the released rejuvenator from the capsules may contribute to a better induction healing effect in the damaging and healing cycles.

## 1. Introduction

In the Netherlands, the concept of zeer open asfaltbeton (ZOAB), which is known as porous asphalt (PA) in the rest of the world, was first applied in 1972 [1]. With a void content above 20%, PA shows advantages in noise reduction, comfortable driving and reduction of splash and spray during rainfall, which has resulted in it being implemented quickly in asphalt pavement design in both the Netherlands and worldwide [2,3,4].

**Figure 1:**
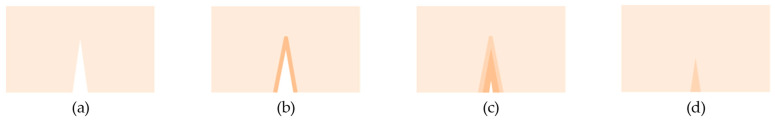
The basic principle of crack healing in asphalt pavement: (**a**) a crack generated in asphalt mastic; (**b**) the ‘mobile phase’ induced at the crack face; (**c**) closure of the crack by the ‘mobile phase’ and (**d**) immobilisation after healing (Hager et al., 2010).

Microcracking is one of the most common early distresses in PA which deteriorates the stone-to-stone contact and can develop into macroscopic damages (e.g., ravelling), and the healing of microcracks is considered as the key factor in delaying or preventing ravelling in PA, therefore extending the lifespan of PA [5]. Consistent with the basic principle of general self-healing materials, the crack healing in asphalt pavement relies on the subsequent generation of a ‘mobile phase’ which gradually results in crack closure during the rest period. Figure 1 illustrates an asphalt crack healing event: when a crack occurs (Figure 1a), the subsequent generation of a ‘mobile phase’ (Figure 1b), triggered either by the intrinsic healing capacity of bitumen or by external stimuli, can heal the crack with the flow of bitumen or mastic (Figure 1c). After crack closure, the previously mobile material is immobilised again, resulting in the regain of mechanical bonding (Figure 1d) [6].

At the early stage of a PA pavement, microcrack healing can be achieved with the intrinsic healing capacity of the bitumen. However, this bitumen-intrinsic healing capacity diminishes with bitumen ageing, which not only reduces the temperature susceptibility of bitumen but also leads to a lower bitumen ductility [7,8]. At that moment, extrinsic healing methods can be employed to induce the ‘mobile phase’ to achieve microcrack healing. The thermally induced healing method heats the asphalt mixture with microwave or induction energy, therefore allowing the bitumen to flow to heal the crack [9,10,11,12,13,14,15]. The embedded rejuvenator encapsulation method offers in situ rejuvenation at the cracking site; as such, the rejuvenated bitumen will regain the intrinsic healing capacity and gradually heal the crack driven by capillary flow [16,17,18,19,20,21,22]. Both methods have been demonstrated to not only improve the crack recovery in asphalt pavement but also increase its fatigue life [23].

**Figure 2:**
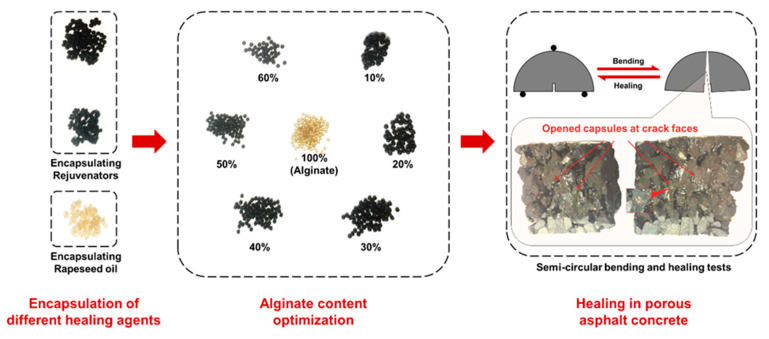
Development of the calcium alginate capsules healing system.

The authors of an earlier study [24,25] have investigated the calcium alginate capsules healing system, which is illustrated in Figure 2. The calcium alginate capsules encapsulating the rejuvenator were prepared and optimized, and their crack healing effect was demonstrated in bituminous materials [24,26]. It was also found that the healing efficiency of the induction heating technique could be largely reduced by asphalt ageing and gradient healing [27,28,29]. Additionally, the induction healing system was introduced to work together with the calcium alginate capsules healing system, and this combined asphalt healing system could not only combine the advantages from both systems but also create synergistic effects, hence offering a better healing prospect [27]. Results from previous findings successfully evaluate the efficiency of each self-healing system in the healing of one major propagating crack; however, the performance of these self-healing systems under cyclic fatigue loadings, especially for the calcium alginate capsules healing system and the combined healing system, is still unknown.

The self-healing performance of an asphalt mixture under fatigue loadings can be investigated using the four-point bending fatigue test (4PB) and the indirect tensile fatigue test (ITF):

- Based on 4PB, Liu et al. [30] evaluated induction healing effect on the fatigue damage in PA. Liu et al. discovered that asphalt beams incorporated with steel wool fibres not only exhibited a higher fatigue resistance in the first 4PB cycle but also gained higher stiffness and showed significantly longer fatigue life when induction heating was introduced during the rest period. Liu et al. indicated that the induction healing rate is highly applied microstrain dependent and the optimum induction heating temperature is 85 °C. Based on these findings, Liu et al. believed that the durability of PA pavement can be improved by induction healing.
- Tabaković et al. [31] employed a 4PB and healing programme to investigate the fatigue damage recovery prospect of alginate fibres in a full asphalt mix. Tabaković et al. found that, after a 20-h healing period at 20 °C, the asphalt beams incorporated with alginate fibres showed a higher stiffness recovery than the controlled beams, indicating that the alginate fibres are a promising approach to improve the self-healing capacity of asphalt pavement. Tabaković et al. further indicated that 4PB is the most suitable test for evaluating the performance of self-healing asphalt.
- The 4PB was also used by Sun et al. [32] to study the fatigue behaviour of self-healing asphalt with melamine urea formaldehyde (MUF) microcapsules. Both the modulus recovery ratio and fatigue life extension ratio were used to evaluate the healing efficiency of MUF microcapsules, and the results indicate that the addition of MUF microcapsules can improve both ratios of the asphalt mixture, thus achieving a better healing performance.
- Menozzi et al. [33] used an ITF and healing programme to examine the induction healing efficiency on an asphalt mixture. The damage and healing in the asphalt mixture were characterized with computed tomography tests. Menozzi et al. reported that the lifetime of Marshall test samples subjected to fatigue damage can be extended with the induction healing.

The findings above show that both 4PB and ITF can be used to characterize the self-healing behaviour of asphalt mixture under fatigue loadings if proper rest periods are introduced based on the built-in extrinsic healing methods.

**Figure 3:**
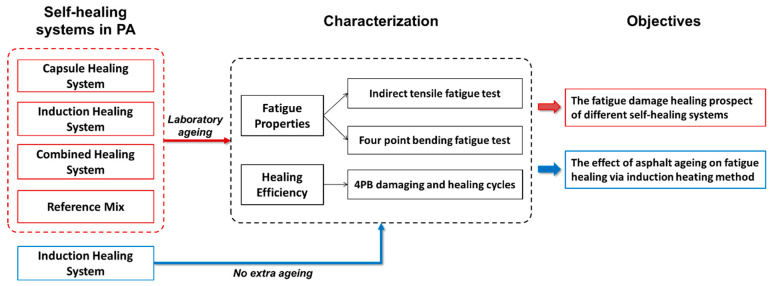
The research methodology of this study.

The main objective of this study focused on the fatigue damage healing prospect of PA mixtures incorporated with various healing systems, namely the capsule healing system, the induction healing system, the combined healing system and a reference mix (without extrinsic healing). The fatigue behaviour of PA with various healing systems was studied using ITF and 4PB. A 4PB damaging and healing programme was carried out to evaluate the fatigue damage healing efficiency of various healing systems, and the fatigue healing index was obtained from the development of the damage rate, which considers changes in both stiffness and fatigue life. Moreover, the influence of asphalt ageing on the fatigue damage healing via the induction heating method was investigated by including a test group of PA mixture (incorporated with the induction healing system) without an extra ageing process. The research methodology is illustrated in Figure 3.

## 2. Materials and Methods

In this section, the built-in healing systems and the preparation method of the PA cylinder and beam specimens are presented. Then, the ITF and 4PB test setups used in this study are introduced, followed by the healing procedure and the damaging and healing programme, which was finally used to evaluate the fatigue damage healing efficiency of different healing systems.

### 2.1. Porous Asphalt Sample Preparation

**Figure 4:**
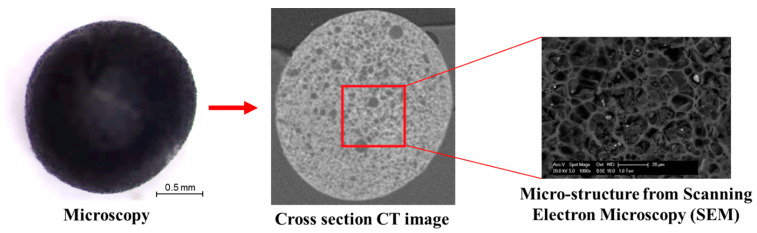
Images of a single calcium alginate capsule. Reproduced with permission from refs. [24,26]. Copyright 2018 Elsevier Ltd.

The calcium alginate capsules and the steel fibres were used to build the self-healing systems in PA. The calcium alginate capsules used in this study were prepared in Microlab, TUDelft, Delft, the Netherlands and the microscopic images in Figure 4 show that the capsules had a diameter of 1.95 mm and a honeycomb-like structure [24,26]. Aiming to improve the conductivity of PA to achieve induction healing, steel fibres were used in the induction healing system and combined healing system. Steel fibres with a density of 7.6 g/cm^3^, an average length of 1.4 mm, a diameter of 40 µm and a resistivity of 7 × 10^−7^ Ω·cm were provided by Heijmans Infra BV, Rosmalen, The Netherlands.

To obtain test specimens for ITF and 4PB, the PA mixture was mixed with a rotating drum mixer and then compacted into slabs with a roller compactor. The materials and mix composition of the PA mixture incorporated with various healing systems can be referred to in a previous study [27]. After compaction, a laboratory ageing process was used to simulate the condition when healing was needed (after years of serving) [27]. Hence, based on the ageing levels and the built-in healing systems, five different PA mixture groups were derived and the detailed group information is presented in Table 1.

Two types of PA slab were fabricated for the study of various healing systems in which Slab_type_1 has the dimensions of 500 × 500 × 50 mm and Slab_type_2 has the dimensions of 600 × 400 × 80 mm. The Slab_type_1 was used for the drilling of cylinder specimens for ITF which had a diameter of 100 mm and a height of 50 mm. The Slab_type_2 was used to produce beam specimens for the 4PB which had dimensions of 400 × 50 × 50 mm.

**Figure 5:**
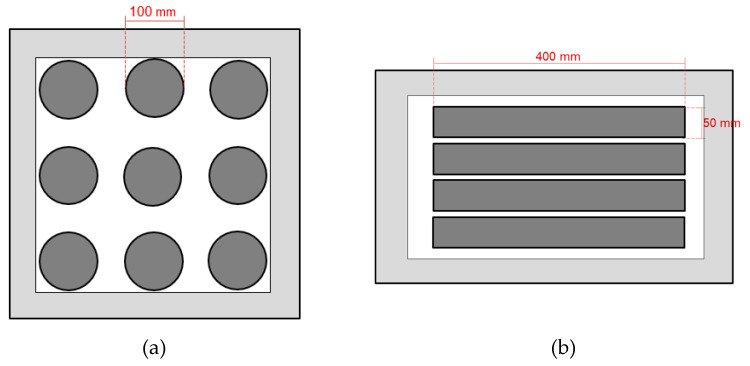
Cylinder and beam samples’ detailed drilling/cutting schematic: (**a**) cylinder samples drilled from Slab_type_1 and (**b**) beam samples cut from Slab_type_2.

Figure 5 shows schematic diagrams of the detailed test sample drilling/cutting process. As shown in Figure 5, a minimum of nine PA cylinders were drilled from Slab_type_1 (Figure 5a), and four PA beams were cut from Slab_type_2 (Figure 5b). During the drilling/cutting process, a 5-cm edge around the slabs (the light grey area) is ignored to avoid edge effect from the compaction process.

### 2.2. Indirect Tensile Fatigue Test

**Figure 6:**
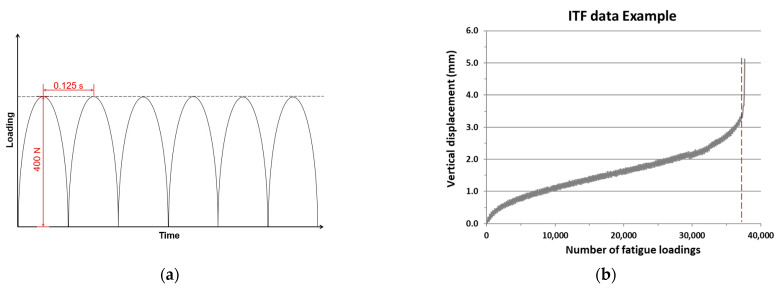
The ITF test: (**a**) the loading configuration schematic and (**b**) ITF test data example, where the red dashed line indicates the fatigue life.

ITF tests aimed to evaluate the fatigue life of a PA sample by recording the total number of continuous loading cycles that the sample can bear, and the loading mode is selected as the stress control. Figure 6a shows the loading configuration schematic for ITF. Following the European standard EN 12697-24, the ITF was carried out by applying a continuous haversine fatigue loading with a peak value of 400 N and the loading frequency of 8 Hz. The ITF was performed in a temperature chamber of 5 °C to avoid permanent deformation upon loadings. The tests were terminated at the point of the full failure and the number of fatigue loadings that led to the sample failure was recorded. Figure 6b shows the data acquired from the ITF, where the red dashed line in the graph shows the maximum number of ITF, which indicates the fatigue life of the test specimen.

### 2.3. Four-Point Bending Fatigue Test

**Figure 7:**
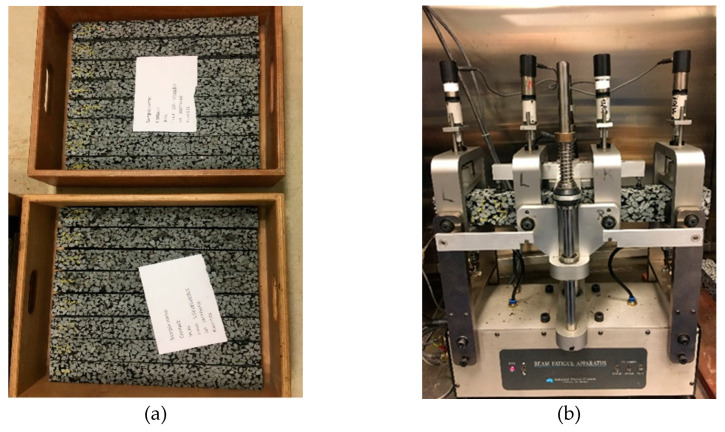

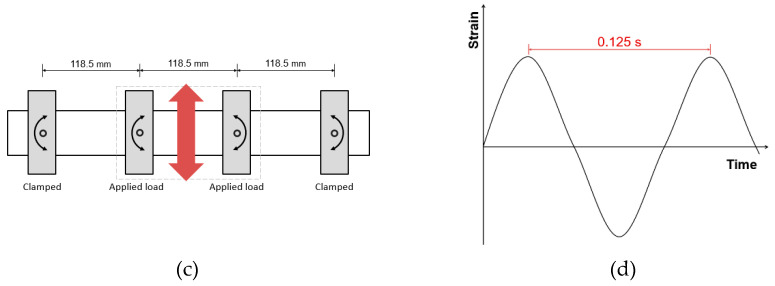
4PB fatigue test: (**a**) beam samples, (**b**) 4PB testing schematic, (**c**) testing setup and (**d**) loading configuration.

During the service life of asphalt pavement, the asphalt layer will be subjected to a high number of bending loads, which leads to fatigue damage, and 4PB is regarded as the most representative laboratory method which can be used to determine the fatigue performance of an asphalt mixture under controlled bending loads [34,35]. In this paper, 4PB aims to investigate the fatigue behaviour of PA beams incorporating various extrinsic healing systems under strain-controlled fatigue loadings. Followed by a rest period, the fatigue damage healing capacity of the PA beams was evaluated. Figure 7 shows the 4PB fatigue test samples, test setup and loading configuration. Figure 7a shows the testing beams kept on a plain wooden board and stored in the storage room at 5 °C. Figure 7b shows the 4PB testing setup where the middle of the beam is subjected to a continuous sine shaped loading by the inner two clamps with strain control. Figure 7c shows the schematic of the 4PB test schematic, and Figure 7d shows the loading configuration in which the loading frequency is 8 Hz. The 4PB fatigue tests were performed in a temperature chamber of 20 °C, and the maximum strain was set as 400 µε. The number of load cycles at the time when stiffness modulus decreases to 50% of its initial level is regarded as the fatigue life of a 4PB fatigue test (EN 12697-24).

**Figure 8:**
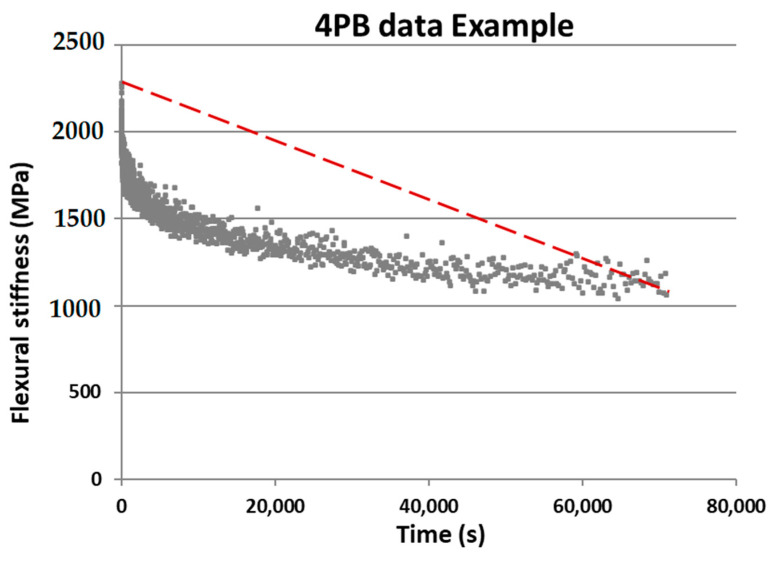
4PB test data example, where the slope of the red dashed line shows the damage rate.

During a 4PB fatigue test, the changes in flexural stiffness of the beam specimen are recorded with the increase of fatigue loadings. Figure 8 shows the data acquired from the 4PB fatigue test, which shows the measured flexural stiffness with time. The red dashed line in the graph shows the average decreasing rate of the flexural stiffness whose slope indicates the damage rate (*D*) of the beam specimen. The damage rate (*D*) in a 4PB fatigue test indicates the average decreasing rate of the flexural stiffness of a beam specimen, which means that a higher damage rate results in a faster damaging process and therefore displays a lower fatigue resistance [34]. The damage rate of a 4PB test considers both the change in stiffness and the time it takes to make this change, which provides a more comprehensive method to illustrate 4PB fatigue behaviour. The following equations can be used for the calculation of the damage rate (*D*):

(1) $$S_{t}=\frac{S_{0}}{2}$$

(2) $$t=\frac{N_{f}}{f}$$

(3) $$D=\frac{S_{0}-S_{t}}{t}=\frac{4S_{0}}{N_{f}}$$

where:

$S_{0}$ is the initial flexural stiffness (MPa);
$S_{t}$ is 50% of the initial flexural stiffness at time *t* (MPa);
t is the time to reach 50% of the initial flexural stiffness (s);
$N_{f}$ is the number of loadings to reach 50% of the initial flexural stiffness;
f is the fatigue loading frequency which is 8 Hz;
D is the damage rate (MPa/s).

The total number of fatigue loadings ($N_{f}$), the flexural stiffness (S) and the damage rate (*D*) are the key parameters that are used to illustrate the fatigue life, stability and fatigue resistance of the beam specimens, respectively, in the 4PB fatigue test.

### 2.4. Healing Procedure

**Figure 9:**
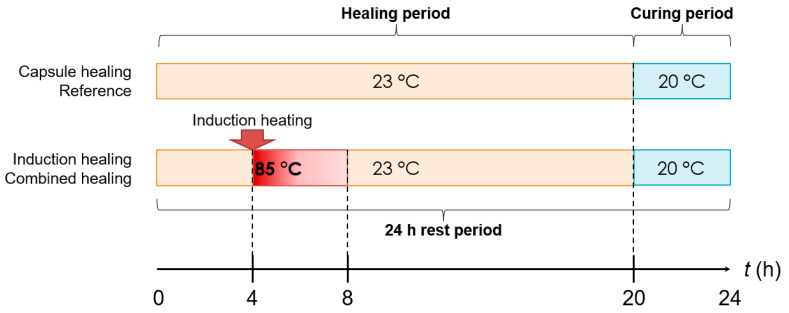
24-h rest period for the healing of various healing systems.

The healing effect of the extrinsic healing technology for self-healing asphalt is largely affected by the rest period as well as the provided environmental conditions, such as time, temperature, humidity, etc. The importance of a rest period in asphalt healing has been proven by the asphalt service life extension in both laboratory testing and field application [36,37]. As such, a proper healing procedure for each healing system needs to be designed. To this aim, a 24-h rest period is designed, which is illustrated in Figure 9. The rest period begins with the 20-h healing period where the majority of healing actions take place by activating the built-in healing system so that the damage healing process is largely accelerated.

Figure 9 shows that, for the healing of damaged samples with the capsule healing system and without a healing system, the whole healing period is conditioned in a temperature chamber at 23 °C on a plain surface. However, for the healing of damaged samples with the induction healing system and the combined healing system, the first 4 h is conditioned at 23 °C to allow the damaged sample to reach the ambient temperature. After that, the induction heating is applied to increase the sample’s surface temperature to 85 °C, followed by 16 h conditioned in the temperature chamber at 23 °C to cool down and be further healed. After the healing period, the sample is cured in a temperature chamber at 20 °C for 4 h to meet the test temperature for the next 4PB round.

**Figure 10:**
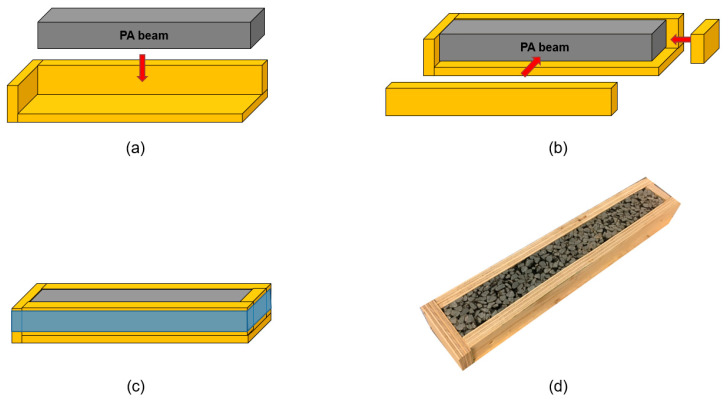
The adjustable wooden box for the confining of beam specimen: (**a**), (**b**) and (**c**) show the schematic confining process for a beam specimen, while (**d**) shows the image of a beam specimen confined in the wooden box.

To avoid permanent deformation during the healing process, constant confinement is created for all samples throughout the 24-h rest period. The 4PB fatigue test is sensitive to the deformations of the tested specimen, and as such, the beam specimens need to be carefully confined to avoid permanent deformation or even loss of particles during induction heating, transportation and the rest period. The adjustable wooden boxes were made to provide the confinement for beam specimens throughout their 24-h rest period. The confining process for beam specimens is presented in Figure 10. Figure 10a shows that the PA beam specimen is placed in the corner of a wooden frame fixed on three sides. Afterwards, a wooden bar is added to cover the front side of the beam, and a piece of wood with a suitable size is placed at the right side of the beam (Figure 10b). Finally, the wooden box is wrapped with tape to ensure that the adjustable two pieces of wood are closely secured against the beam sample (Figure 10c). Figure 10d shows the image of a beam specimen confined in the wooden box.

### 2.5. Damaging and Healing Programme

**Figure 11:**
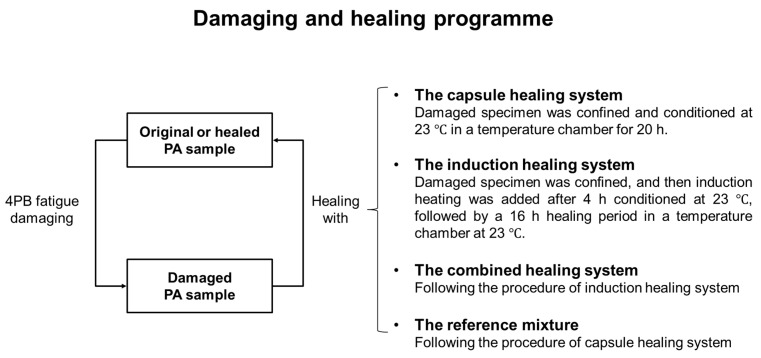
The damaging and healing programme for PA samples with various healing systems.

To evaluate the fatigue damage healing efficiency of PA samples from each mixture group, a damaging and healing programme was designed based on 4PB, which is illustrated in Figure 11. First, the initial property of the testing sample was measured by 4PB. Then, the 24-h rest period was provided based on the built-in healing system, therefore completing a damaging and healing cycle. The damaging and healing cycle(s) continued until the testing sample fully failed (fractured into two parts).

The fatigue healing index is used to illustrate the fatigue healing effect of a PA mixture. The damage rate (D) acquired from the 4PB fatigue tests is used to characterize the durability of a beam specimen under fatigue loadings, and a higher damage rate refers to a lower performance in the PA durability. The fatigue healing index (FHI) can be calculated with the following equation [34]:

(4) $$FHI=\frac{C_{x}}{C_{1}}\times 100\%$$

where:

FHI is the fatigue healing index (%);
$D_{1}$ is the initial damage rate (MPa/s);
$D_{x}$ is the damage rate measured from the *x* test cycle (MPa/s).

## 3. Results and Discussion

### 3.1. The Fracture Faces of PA Mixture Containing Capsules

**Figure 12:**
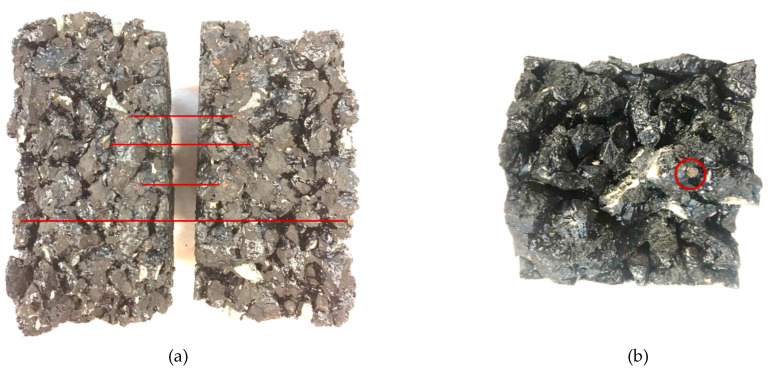
The opened capsules on fracture faces after fatigue loadings: (**a**) fracture faces of a cylinder specimen after ITF and (**b**) fracture face of a beam specimen after 4PB damaging and healing tests.

In the previous study, the calcium alginate capsules were found in two pieces at the fracture faces of the PA mixture sample after the semicircular bending test, demonstrating that these capsules can be opened by the crack propagation thus releasing the rejuvenator. Similar phenomena were observed on the fracture faces of the PA mixture containing capsules after the fatigue test. Figure 12 shows the opened capsules on the fracture faces of the PA samples after fatigue loadings. Although the fracture faces were crushed upon fatigue loadings, broken capsules can be observed on the fracture faces of a cylinder specimen after ITF (Figure 12a) and a beam specimen after 4PB damaging and healing tests (Figure 12b). This finding indicates that the calcium alginate capsules encapsulating the rejuvenator embedded in the PA mixture can be opened upon fatigue loadings, potentially from vehicles, which means that this capsule healing system is qualified as an in situ rejuvenator delivery mechanism to achieve damage self-repair in the long-term service life of PA pavement.

### 3.2. Indirect Tensile Fatigue Results

**Figure 13:**
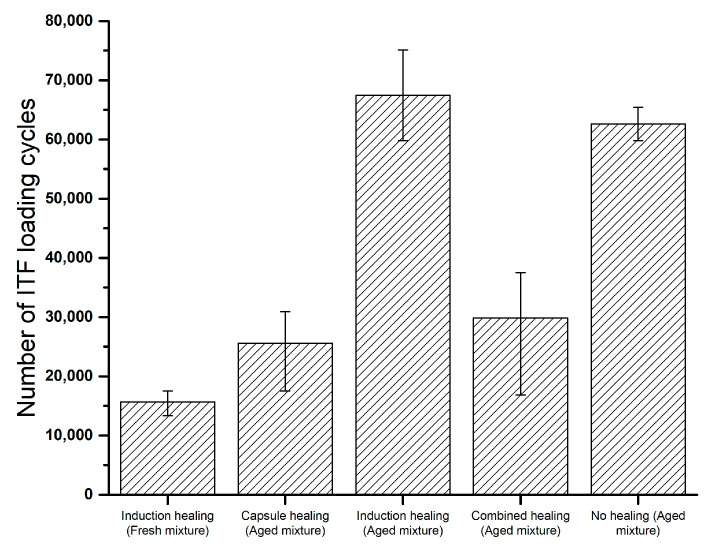
Indirect tensile fatigue results of cylinder specimens from all PA mixture groups.

In the ITF test, the ITF fatigue life of a PA sample is illustrated by the number of loadings that leads to failure. It is also noted that induction heating or a rest period was not applied throughout the continuous fatigue loadings. Figure 13 presents the indirect tensile fatigue test results for all PA mixture groups. The PA samples without laboratory ageing show the least fatigue life, which means that the laboratory ageing process improves the ITF fatigue life, and similar findings were reported by other researchers [38,39]. This might be because the ageing increases the stiffness of PA samples, which improves the samples’ resistance to deformations under fatigue loadings.

The ITF test results also indicate that the incorporation of the induction healing system can extend the ITF fatigue life of PA samples, which can be seen from the results of the aged mixture groups between induction healing and no healing and between combined healing and capsule healing. This might be due to the reinforcing effect from steel fibres, which was also found in the indirect tensile stiffness of these PA samples from the previous study [27]. A similar ITF fatigue life extension effect of the steel fibres was also reported by Liu [40].

However, the PA samples with embedded capsules showed much less ITF fatigue life, which indicates that the presence of calcium alginate capsules will reduce the ITF fatigue life of the cylinder specimens under continuous fatigue loadings. This might be due to the released rejuvenator, either from the opened capsules by microcracking or from being squeezed out by the fatigue loadings, which could develop in two ways:

1. The released rejuvenator worked. The encapsulated rejuvenator released from capsules and softened the aged binder to reduce the stiffness of the PA sample and, finally, resulted in a reduction in ITF fatigue life that behaved like the PA samples with a fresh mixture;
2. The released rejuvenator did not work. The rejuvenator released upon continuous fatigue loadings but was not able to diffuse into the aged binder at a low temperature (5 °C). In this case, the rejuvenator would be located at the damage site in a liquid phase which might cause slippage, and this could be amplified under indirect tensile fatigue loadings.

### 3.3. PB Damaging and Healing Test Results

#### 3.3.1. Effect of Asphalt Ageing on Induction Healing

**Figure 14:**
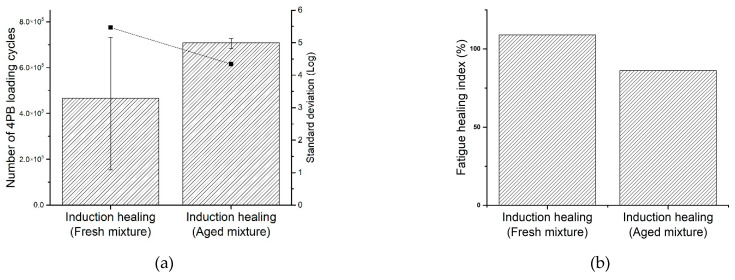
Four-point bending fatigue test results for the induction healing system in PA mixture with different ageing levels: (**a**) total number of 4PB fatigue loading cycles and (**b**) the fatigue healing index.

Asphalt ageing makes the binder stiffer, and this will result in a reduction of the asphalt healing effect with the induction heating method (Xu, Shi et al., 2018). In this study, the influence of asphalt ageing on the fatigue damage healing with the induction heating method was investigated, and the results are presented in Figure 14. Figure 14a shows that the aged PA mixture has a longer fatigue life than the fresh PA mixture in the damaging and healing test cycles, which is similar to the ITF test results, while the difference is not that significant.

Figure 14b shows that the induction healing system has a higher fatigue healing index on the fresh mixture, which is 108.9%. This means the stiffness decreasing rate of the beam specimens with fresh mixture became even slower after the induction healing process, while the fatigue healing index for the aged mixture is 86.2%. As a result, the induction heating approach has a promising fatigue damage healing effect especially on a fresh mixture, and this healing effect is better than the PA mixture treated with the laboratory ageing process. Although the longer fatigue life of the aged beams is confusing, the fatigue healing index calculated from the damage rate successfully illustrates the decreasing of induction healing efficiency due to asphalt ageing.

#### 3.3.2. The Healing Effect of Four Asphalt Healing Systems

**Figure 15:**
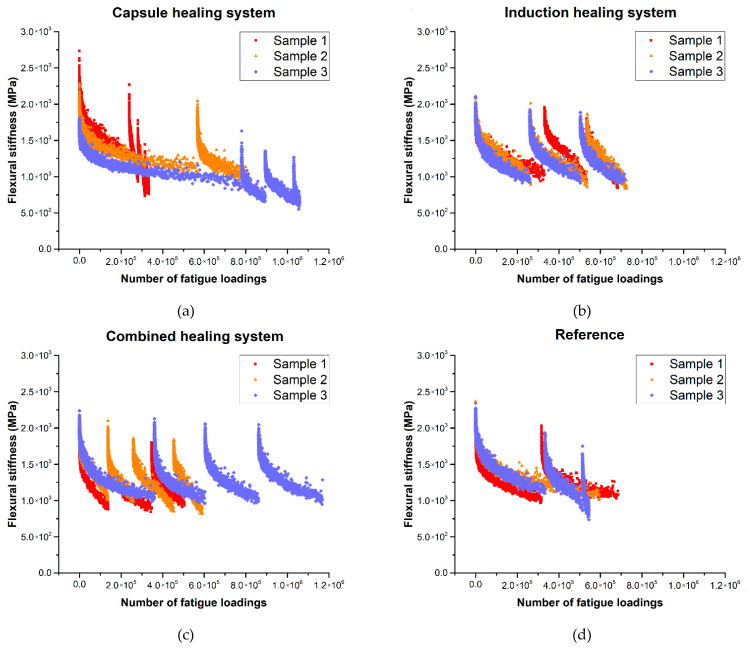
Four-point bending fatigue test results for the aged mixture with various healing systems: (**a**) the capsule healing system, (**b**) the induction healing system, (**c**) the combined healing system and (**d**) the reference mix.

Figure 15 presents the 4PB fatigue test results for various healing systems incorporated in the aged PA mixture, which illustrates the fatigue behaviours of each healing system in the damaging and healing cycles.

Figure 15a shows the 4PB fatigue test results for the capsule healing system. Damages that took place in the first 4PB fatigue test were recovered in the rest period, which allowed the beam specimen to regain a part of the lost flexural stiffness. However, due to the softening effect from the released rejuvenator, samples with the capsule healing system showed a continuous reduction in both 4PB fatigue life and flexural stiffness with the increase of testing cycles. Furthermore, the beam specimens with the capsule healing system showed a large variety in the 4PB fatigue life results, which indicates that the calcium alginate capsules have an unstable impact on the total 4PB fatigue life tested from the 4PB fatigue test cycles.

Figure 15b shows the 4PB fatigue test results for the induction healing system. The induction healing system showed more stable healing than the capsule healing system, which lies in the recovery of both flexural stiffness and 4PB fatigue life. The results acquired from the third fatigue test cycles could still have an average maximum flexural stiffness of 1845 MPa, which is more than 85% of the average initial stiffness, which is 2080 MPa. Furthermore, the three beam specimens with induction healing systems showed a very similar 4PB fatigue life, as opposed to a large variety for the specimens with the capsule healing system.

Figure 15c shows the 4PB fatigue test results for the combined healing system in which the fatigue behaviours of both the capsule healing system and induction healing system are found. The combined healing system showed an effective recovery on the flexural stiffness from the rest period and a significant 4PB fatigue life extension effect. However, the combined healing system leads to different 4PB fatigue life extensions of the three beam specimens, which is similar to the capsule healing system.

For the reference beams without healing systems, the fatigue damage healing actions still took place during the 24-h rest period, which showed a notable extension of 4PB fatigue life as well as a recovery of stiffness for Sample 2 and Sample 3 (Figure 15d). However, the reference beams have a lower number of average possible healing cycles, and the healing effect is limited compare to beams with a built-in healing system.

**Figure 16:**
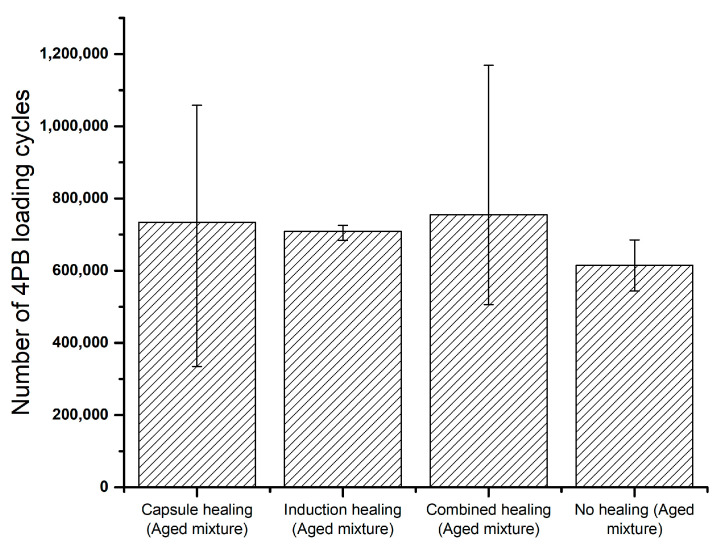
Number of 4PB fatigue loadings leading to failure of all beam specimens.

Figure 16 shows the summary of the total number of 4PB fatigue loading cycles which leads to the failure of the beams. In Figure 16, among the aged PA samples with various healing systems, beams from the reference group showed the lowest number of loading cycles, which means that the incorporation of these healing systems may result in an increased 4PB fatigue life.

It is also indicated that the PA samples with the combined healing system have the highest fatigue life; however, the data dispersion is large, which is also found in the results for the capsule healing system. It might be the localised rejuvenation effect from the embedded capsules that softened the aged material at the damage site, therefore slowing down the development of microcracks under the repeated fatigue loadings where the rejuvenation took place. As a result, the fatigue damage healing with the capsule healing system is determined by the distribution of the opened capsules, whereby leading to the variety in 4PB fatigue life.

**Figure 17:**
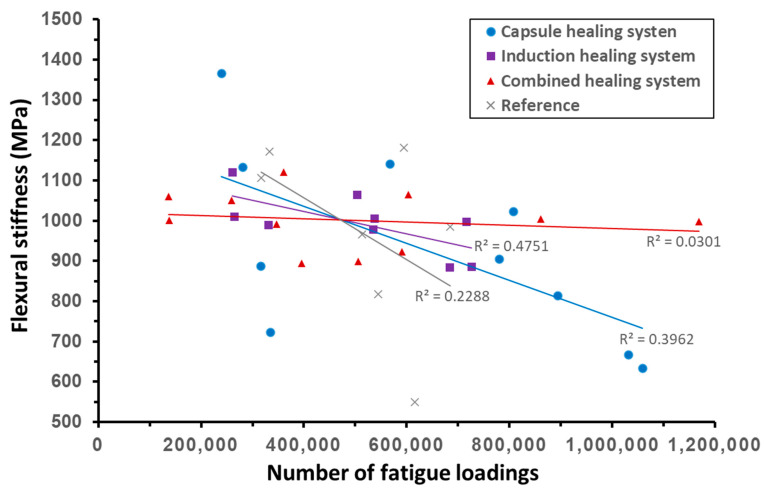
Development of flexural stiffness for the various healing systems.

Figure 17 summarises the developments of flexural stiffness with the 4PB fatigue loadings for all the aged beams. Despite some scattered results from the capsule healing system and the reference group, the general trend for the stiffness of beam specimens developed throughout the damaging and healing programme reflects the stiffness stability of a healing system. The flexural stiffness of the combined healing system decreased slowly, followed by the induction healing system and the capsule healing system, while the reference group showed much faster decrease of flexural stiffness under fatigue loadings. These findings can be better illustrated with the slopes of trendlines in Figure 17, which decrease from the combined healing system (red) to the induction healing system (purple), the capsule healing system (blue) and the reference group (grey). Hence, the combined healing system shows an advantage in stiffness recovery under fatigue loadings over the other healing systems.

**Figure 18:**
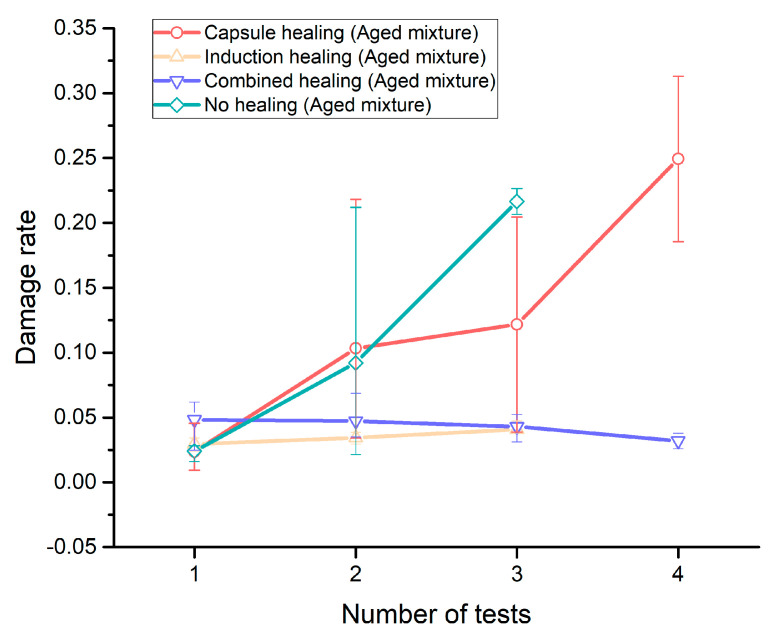
The damage rate acquired from each 4PB damaging and healing cycle.

For all aged PA mixtures, the damage rate acquired from each 4PB damaging and healing cycle is presented in Figure 18. The capsule healing with the aged mixture group showed an increasing trend in damage rate during the 4PB fatigue test cycles, which means that the beams incorporated only with the capsule healing system more easily lost the regained stiffness in the following fatigue test cycles. Compared to the reference (no healing) group, the capsule healing system showed two advantages in fatigue damage recovery: much lower damage rate after two rest periods and one extra potential healing cycle. This might be because the released rejuvenator showed a more significant damage healing effect after two rest periods, and then stimulated the healing of microcracks in beam specimens to be able to conduct the fourth 4PB fatigue test before failure.

The damage rate for the induction healing (aged mixture) group slightly increased with the increase of fatigue test cycles (Figure 18); however, it is much lower than the capsule healing group and the reference (no healing) group in the second and the third 4PB fatigue test cycles, which indicates the advantage of the induction healing system in fatigue damage healing.

The combined healing system showed a more stable and durable healing effect than the induction healing system, which not only showed a decreased trend in damage rate but also survived four 4PB fatigue test cycles. As a result, the combined healing system demonstrated the best performance in fatigue damage healing in comparison to the single extrinsic healing systems (capsule healing and induction healing) and the intrinsic asphalt healing.

### 3.4. Fatigue Healing Index

**Figure 19:**
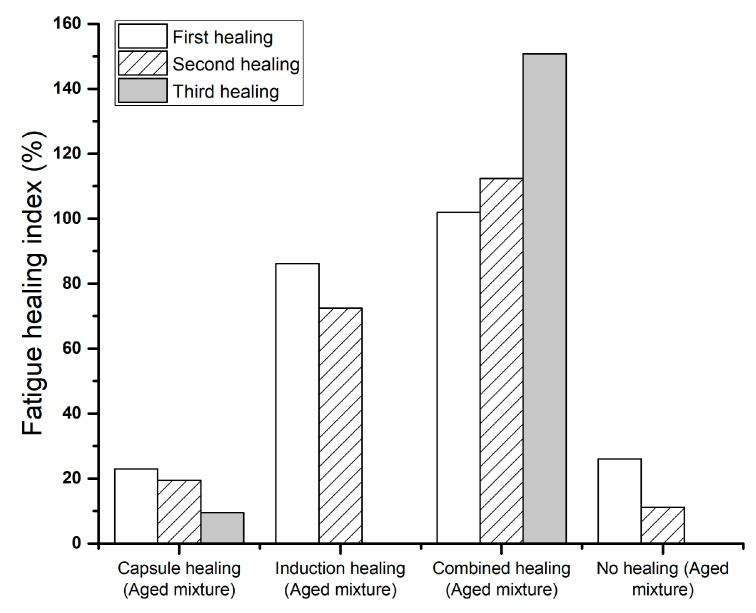
The fatigue healing index for various healing systems.

The fatigue healing index (FHI) acquired from the damaging and healing programme for all four healing systems is shown in Figure 19. Compared to the no healing group, the capsule healing group showed a lower FHI in the first healing, but a higher FHI in the second healing, and could achieve effective healing for three cycles. This indicates that the capsule healing system can improve the healing capacity of an aged PA mixture and achieve a more durable fatigue behaviour in the 4PB test series. The induction healing (aged mixture) group has a much higher fatigue healing index than the capsule haling group in the first two healing events, which indicates that induction heating has a much better fatigue damage healing effect than capsule healing. However, the induction healing system could not provide effective healing for the third time. When the capsule healing system and induction healing system are combined, the fatigue damage healing effect is significantly improved (Figure 19). In contrast to the capsule healing system and the induction healing system, the combined healing system shows a much higher FHI during all testing cycles, and these values even increase after every healing event. A possible explanation is the gradual healing effect from the calcium alginate capsules whose compartmented rejuvenator is gradually released upon the fatigue loadings, so that the induction healing effect is enhanced due to aged binder rejuvenation time after time. Finally, the terrific fatigue damage healing effect from the combined healing system is demonstrated.

## 4. Conclusions

This paper presents a study on the fatigue damage healing prospects of porous asphalt incorporated with four different self-healing systems. The following conclusions can be drawn:

- The ITF test results indicate that the calcium alginate capsules show a negative effect under the stress-controlled fatigue life, which might be due to the fact that the released rejuvenator from the capsules softens the aged material and results in higher deformations under stress-controlled fatigue loadings. It could also be possible that the released rejuvenator can hardly diffuse and rejuvenate at 5 °C under continuous loadings.
- The rest period plays an important role in determining the healing effect of all asphalt self-healing systems. For the capsule healing system, the curing temperature should not be too low. For the induction healing system, stable confinement is recommended to avoid permanent deformation under a high temperature (85 °C).
- Induction heating provides stable stiffness recovery and a reliable 4PB fatigue life extension effect in the long-term damaging and healing cycles. This finding agrees with the conclusions from existing research [40].
- The beam specimens with the capsule healing system showed the longest fatigue life in the damaging and healing cycles, which might because the rejuvenator released from the capsules improves the healing capacity of the aged materials, thus resulting in a more durable fatigue behaviour in the test series. The scattered results might be caused by the random capsule distribution which determines whether the healing takes place on the damage site or not.
- The combined healing system showed the best performance in fatigue life, stability and fatigue resistance, which points to the healing effect from the calcium alginate capsules whose compartmented rejuvenator is gradually released upon the fatigue loadings, so that the induction healing effect is enhanced due to aged binder rejuvenation time after time. As such, the terrific fatigue damage healing effect from the combined healing system is demonstrated.
- Additionally, by comparing the results between the induction healing system on the aged mixture and the fresh mixture, it turns out that ageing actually contributes to longer fatigue life but results in a higher damage rate and lower healing index.

Among all four asphalt healing systems, the combined healing system is demonstrated to be the most promising method to extend the service life of porous asphalt pavement based on the results from the four-point bending fatigue tests. For future research, it is strongly recommended to further optimize the capsule healing system and the combined healing system by reducing, for example, the calcium alginate capsules’ diameter to improve distribution in the asphalt mixture. This will generate progress towards field applications of the combined self-healing system in asphalt pavement.

## Figures and Tables

**Table 1:** PA mixture group information.

PA Mixture Group Name	Laboratory Ageing	Built-In Healing Systems
Induction healing (fresh mixture)	No	Induction
Capsule healing (aged mixture)	Yes	Capsules
Induction healing (aged mixture)	Yes	Induction
Combined healing (aged mixture)	Yes	Capsules and induction
No healing (aged mixture)	Yes	None

## Data Availability

The raw/processed data required to reproduce these findings cannot be shared at this time as the data also form part of an ongoing study.
